# Supplementary material for: Complexome profiling of the Chlamydomonas psb28 mutant reveals TEF5 as an early PSII assembly factor
Source: Plant Cell. 2025 Mar 17;37(6):koaf055. doi: 10.1093/plcell/koaf055 (PMC12164590; doi:10.1093/plcell/koaf055)
Supplement: koaf055_Supplementary_Data [file koaf055_supplementary_data.zip › psb28_interactive.zip!/plots/intensity/qualityPlots/peptides/CPLD29_Cre02.g088500.t1.1.html]

### Cre02.g088500.t1.1

Trivial name: CPLD29  
SeqCoverage [median]: 14.30  
Mass: 52.90 kDa  
  
 

| Sequence | MissedCleavage | Mass [Da] | wtIntensity | muIntensity | Score |
| --- | --- | --- | --- | --- | --- |
| AAPEPIPEDAAAASAAAAASGK | 0 | 1935.95 | 0.00 | 4001.90 | 56.53 |
| DLVESSVKPGSSALK | 1 | 1515.81 | 636.00 | 0.00 | 40.55 |
| FVIEDVTSGDPR | 0 | 1333.65 | 742.64 | 2109.41 | 102.06 |
| LLGALTPLIR | 0 | 1065.69 | 513.64 | 0.00 | 79.91 |
|  |  |  | 1892.3 | 6111.3 |  |
